# Wandering Spleen Torsion: A Diagnostic Challenge

**DOI:** 10.7759/cureus.53552

**Published:** 2024-02-04

**Authors:** Malak Eleiwi, Yazid Atatri, Omar Younis, Jehad Zuhd, Ahmed Awadghanem, Ahmad Qashoo, Suha Sholi, Samer Bustame

**Affiliations:** 1 Medicine, An-Najah National University Hospital, Nablus, PSE; 2 Medicine, Faculty of Medicine and Health Sciences, An-Najah National University, Nablus, PSE; 3 Anesthesia, An-Najah National University Hospital, Nablus, PSE; 4 General Surgery, An-Najah National University Hospital, Nablus, PSE; 5 Pediatric Surgery, An-Najah National University Hospital, Nablus, PSE

**Keywords:** acute abdomen, case report, gastroenteritis, torsion, splenopexy, splenectomy, wandering spleen

## Abstract

Wandering spleen, or hypermobile spleen, arises from the elongation or maldevelopment of the spleen's suspensory ligaments. This condition is a rare clinical entity, primarily affecting children, with a higher prevalence among adult females in the active reproductive age group. Manifestations may include an asymptomatic abdominal mass or intermittent abdominal discomfort due to the torsion and subsequent spontaneous detorsion of the spleen. This case report details the presentation of a 14-year-old female initially misdiagnosed as having gastroenteritis who later experienced acute abdomen. Subsequent ultrasonography and computed tomography scan revealed splenic torsion, confirmed during exploratory laparotomy, which demonstrated an infarcted spleen. The definitive therapeutic intervention was a total splenectomy. This clinical entity should be taken into account in the differential diagnosis of acute abdominal pain in order to aid in early diagnosis and management. This could allow us to avoid splenectomy whenever possible and instead do splenopexy, especially in pediatric cases, as the spleen plays a crucial role in the reticuloendothelial system.

## Introduction

Abdominal pain is one of the most common complaints in children, and it can be challenging to diagnose due to the variety of possible underlying causes [1]. In most cases, abdominal pain is benign and self-limiting, stemming from various factors including gastrointestinal infections, dietary issues, constipation, and inflammatory conditions. Less common but more serious conditions may also be responsible; therefore, physicians should maintain a high index of suspicion for uncommon and potentially life-threatening conditions in children with abdominal complaints that may require urgent evaluation and surgical intervention, such as adhesions, appendicitis, or other surgical cases [2].

Wandering spleen is a rare condition that results from elongation or maldevelopment of the spleen’s suspensory ligaments, which hold the spleen in its normal anatomical position in the left upper quadrant of the abdomen [3]. It can present with a torsion of its pedicle causing an acute abdomen, or it may be an incidental finding presenting as an asymptomatic, palpable abdominal mass. The conventional therapeutic approach involves the fixation of the spleen (splenopexy) unless there is an infarction and there is no evidence of blood flow to the spleen after detorsion, in which case splenectomy should be considered [4]. Due to its rarity as well as the nonspecific nature of its clinical presentation, it has been a diagnostic challenge for clinicians [5].

While extensively documented in adult literature, the occurrence of wandering spleen has been increasingly reported in the pediatric population in recent times, and knowledge about this pathology originates mainly from individual case reports [6]. Even with the advancement of available diagnostic techniques, this rare and elusive disorder continues to be misdiagnosed, emphasizing the necessity for a heightened level of suspicion to establish the correct diagnosis [7].

We report a case of a young girl, initially misdiagnosed as having gastroenteritis, who presented with torsion of a wandering spleen, treated by splenectomy due to infarction. The patient's parents were provided with a detailed explanation of the study objectives, and they voluntarily agreed to document their child’s case by signing the informed consent form.

## Case presentation

A 14-year-old female patient sought medical advice at an outpatient clinic due to symptoms of nausea and vomiting persisting for two days, with a normal physical examination, and was initially managed as a case of gastroenteritis with no improvement, as reported by the parents. The following day, the patient was admitted to the surgical ward from the emergency department after presenting with sudden, severe epigastric and lower abdominal pain, which had persisted for nearly one day and was associated with vomiting and fever. She had no recent history of trauma. She was taking oral iron supplements for iron deficiency anemia. Three months prior, she underwent a laparoscopic cholecystectomy for acute calculous cholecystitis. There was no significant family history of a similar condition.

On admission, her vital signs included a heart rate of 95 beats per minute, a blood pressure of approximately 107/69 mmHg, and a temperature of 36.8°C axillary. Physical examination revealed a soft, lax abdomen overall, except for tenderness and a fullness sensation in the lower abdominal region, suggestive of a palpable mass. Other systemic examinations, including neurology, cardiology, and respiratory examination, were normal.

Routine laboratory testing indicated a white blood cell count of 18.7 k/µl, platelets of 337 k/µl, hemoglobin of 10.9 g/dL, and a C-reactive protein level of 14.3 mg/L. Urine analysis revealed many red blood cells and white blood cells. Table 1 shows the initial laboratory results upon presentation.

**Table 1:** Laboratory results upon admission. CBC: Complete blood count, AST: Aspartate aminotransferase, ALT: Alanine transaminase, CRP: C-reactive protein, HPF: High power field.

Parameter	Result	Reference range
CBC with differential		
White blood cell count	18.76	4 - 9 k/µl
Neutrophil %	84	50% - 70%
Lymphocyte %	9.4	18% - 42%
Hemoglobin	10.9	11.1 - 15.7 g/dL
Platelets	337	140 - 450 k/µl
Amylase, serum	112	28 - 100 µl
Lipase, serum	29	13 - 60 µl
AST	12.6	13 - 35 µl
ALT	10.9	10 - 28 µl
CRP (Quantitative)	14.3	0 - 5 mg/L
Urine analysis and microscopic examination		
Urine appearance	Turbid	Clear
Urine blood	+3	Negative
Bilirubin	Negative	Negative
Protein	+3	Negative
Nitrate	Negative	Negative
Ketones	+3	Negative
Glucose	Negative	Negative
pH	6	6 - 8
Specific gravity	1.015	1.005 - 1.030
Leukocyte esterase	+2	Negative
Red blood cells	Many	0 - 3 / HPF
White blood cells	Many	0 - 5 / HPF
Bacteria	Many	Negative

**Figure 1:**
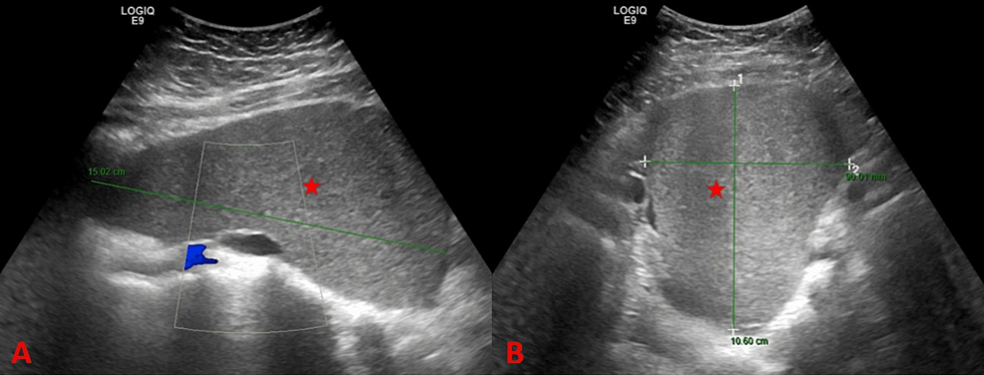
Ultrasound of the patient at the time of admission. A and B: Ultrasound of the lower abdomen and pelvis, showing a large mass occupying the lower abdomen and pelvis with echogenicity similar to spleen (red star).

An ultrasound was ordered due to physical examination findings, which revealed a comma-shaped, large mass occupying the lower abdomen and pelvis, measuring 10.6 x 9 x 15 cm, with echogenicity similar to that of the spleen and without vascular supply, causing compression on pelvic structures. Additionally, it showed the absence of the spleen from its normal location (Figure 1).

**Figure 2:**
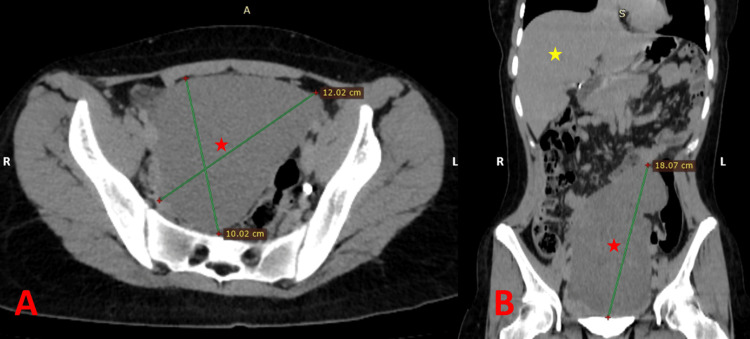
CT scan of the patient at the time of admission. A and B: CT of the abdomen and pelvis, revealing the appearance of the wandering spleen in the lower abdomen and pelvic area in the axial and coronal sections, respectively (the red star represents the spleen and the yellow star represents the liver).

Based on these findings, an abdominal/pelvic computed tomography (CT) scan with intravenous contrast was performed, confirming the diagnosis of an infarcted wandering spleen. The CT scan revealed an enlarged spleen measuring 18 x 12 x 10 cm, with no enhancement, located in the pelvic region; additionally, the distal segment of the splenic artery was not enhanced, with multiple congested veins at the hilum (Figure 2).

**Figure 3:**
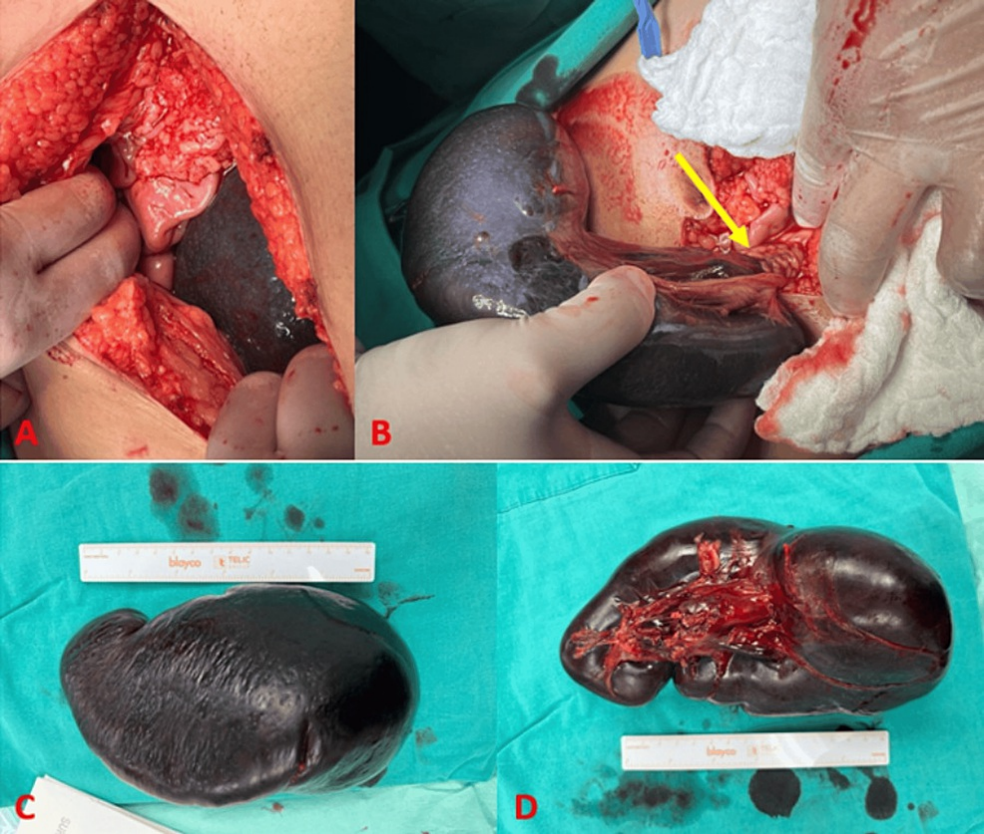
Intraoperative findings. A: Infarcted spleen located in the lower abdomen; B: Enlarged spleen showing the twisted pedicle at the hilum (yellow arrow); C and D: Congested and ischemic spleen (16 x 11 x 6 cm in size) after splenectomy.

The diagnosis of an infracted wandering spleen was established, so the decision to have an urgent splenectomy was made after explaining to the patient’s parents that it was the only treatment option and taking the consent form. A total splenectomy was performed, under general anesthesia, through a midline exploratory laparotomy, and a vacuum drain was inserted in the pelvis. Intraoperatively, the spleen was observed in the lower abdomen. It was found to be infarcted due to twisting around its pedicle, and it was freely mobile. All splenic ligamentous attachments were completely absent (Figure 3). The infarcted spleen was sent for histopathology.

The patient had an uneventful recovery from anesthesia and was transported to the surgical ward postoperatively. The patient was discharged on the fourth day of admission without any postoperative complications. She received recommended vaccines for splenectomy patients, which include *Haemophilus influenzae, Meningococcal, *and *Pneumococcal* vaccines. The patient had uneventful follow-ups with a normal physical examination after 2 weeks and 5 months after the surgery.

Subsequent histopathology confirmed the diagnosis of an infarcted splenic torsion, revealing a congested and hemorrhagic spleen (16 x 11 x 6 cm) with multiple thrombosed blood vessels.

## Discussion

Wandering spleen is an extremely uncommon condition, occurring in less than 0.2% of the population. It predominantly affects children, accounting for one-third of cases, especially those under 10 years old, and shows a higher incidence in females after the first year of life [4].

The etiology of this disorder may be congenital or acquired. The congenital form is due to the faulty development of the dorsal mesogastrium during embryonic life, which results in the absence or underdevelopment of one or more of the ligaments that keep the spleen in its normal anatomical position in the left upper quadrant of the abdomen [8,9]. In contrast, the acquired form is more common in women who have had multiple pregnancies, as the ligaments that secure the spleen tend to become lax [10]. As a result, the spleen in these people can be discovered in unusual abdominal or even pelvic positions. Increased mobility of the spleen, due to laxity in the splenic ligaments and the abnormal length of splenic vessels, may increase the susceptibility to splenic torsion [9].

The clinical presentation of a wandering spleen can manifest across a broad spectrum. Frequently, it presents as an asymptomatic intra-abdominal mass incidentally discovered during routine physical or radiological examinations. However, it may also present with a combination of mass and subacute abdominal complaints. In some instances, acute abdominal findings can occur, primarily attributed to splenic torsion [5,11]. In this particular case, the patient reported pain in both the epigastric and lower abdominal regions, accompanied by nonspecific gastrointestinal complaints that were initially misdiagnosed as gastroenteritis. This misdiagnosis is notable when contrasting the symptoms of gastroenteritis in children, which typically encompass diarrhea, vomiting, and generalized abdominal pain [12]. In contrast, the localized and intense abdominal pain observed in this case aligns more closely with the clinical presentation associated with wandering spleen torsion. This highlights the importance of careful consideration and differentiation of symptoms to avoid diagnostic pitfalls in cases of rare abdominal conditions.

Splenic torsion is the most common complication of wandering spleens, which was identified in this case presentation. A torsion of the spleen can be acute or chronic. Acute splenic torsion, which presents as an acute abdomen, can be misdiagnosed as peritonitis, acute appendicitis, twisted ovarian cysts, or intestinal obstruction [13]. Chronic torsion might manifest as an abdominal mass located in any quadrant with intermittent abdominal discomfort because of torsion and spontaneous detorsion of the spleen [5].

Medical imaging plays a crucial role in the diagnostic evaluation of wandering spleen. Among the array of diagnostic modalities, ultrasonography, and abdominal computed tomography scans emerge as integral tools, providing valuable insights by revealing the absence of the spleen in its conventional anatomical location. Instead, these imaging techniques discern a comma-shaped structure situated in alternative abdominal or pelvic regions [14], as exemplified in our reported case, which showed typical CT findings of a wandering spleen with a torsion of its pedicle and a complete infarction. The findings were confirmed during surgery and subsequent histopathological examination. Otherwise, routine laboratory testing and urinalysis alone may be misleading for the diagnosis of the rare causes of acute abdomen, like torsion of the wandering spleen, as this case showed abnormal urine analysis that was not the actual cause of the presenting symptoms. The contrasting observations in the literature, where normal urine analysis is typically reported in different cases of wandering spleen torsion in various locations [15,16], further underscore the need for a comprehensive approach of integrating advanced imaging techniques to ensure a more accurate and timely diagnosis in uncommon cases such as torsion of the wandering spleen.

Surgical intervention appears to be the preferred approach for managing cases of a wandering spleen [7]. It includes two primary modalities: splenopexy and splenectomy. Whenever possible, efforts should be made to preserve the spleen, as its physiological significance is well-established as a crucial component of the reticuloendothelial system, playing a vital role in antigen clearance, particularly for encapsulated organisms like *Haemophilus influenzae, Meningococcal, *and *Pneumococcal* bacteria [7]. The selection between these therapeutic interventions depends on the clinical circumstances. In instances where the wandering spleen is deemed viable, particularly in pediatric cases, splenopexy is the favored course of action, aiming to preclude potential complications in the future [3]. However, for individuals with a wandering spleen experiencing complications such as rupture, hemorrhage, abscess, splenomegaly causing mass effect, or when faced with acute torsion resulting in splenic infarction and the absence of viable spleen parenchyma, the recommended therapeutic approach is splenectomy. This procedure can be performed using either an open or laparoscopic technique, as preserving the spleen is deemed unattainable [4]. In this patient, the urgent need for a laparotomy and splenectomy was prompted by the presence of splenic infarction coupled with spleen enlargement. Following a splenectomy, encapsulated organism vaccines (*Haemophilus influenzae, Meningococcal, *and *Pneumococcal* vaccines) and annual influenza vaccinations must be administered either on the day of discharge or day 14, whichever comes first, in adherence to the splenectomy vaccination guideline [4,17].

## Conclusions

In conclusion, the wandering spleen presents a diagnostic challenge due to its nonspecific symptoms, ranging from mild discomfort to acute abdomen. Surgical intervention, preferably splenopexy, is recommended if the spleen is viable; however, extensive necrosis may necessitate an open splenectomy. Recognition of wandering spleen torsion is crucial in cases of acute abdominal pain, highlighting the need for comprehensive diagnostic approaches incorporating advanced imaging. Our case emphasizes the importance of including torsion of a wandering spleen in the differential diagnosis for acute abdominal pain, ensuring an accurate and timely diagnosis aimed at spleen preservation.

## References

[1] Marin JR, Alpern ER (2011). Abdominal pain in children. Emerg Med Clin North Am.

[2] Kim JS (2013). Acute abdominal pain in children. Pediatr Gastroenterol Hepatol Nutr.

[3] Jude NN, Onochie NC (2015). Torsion of a wandering spleen. A rare cause of acute abdomen. Saudi Med J.

[4] Wang Z, Zhao Q, Huang Y (2020). Wandering spleen with splenic torsion in a toddler: a case report and literature review. Medicine (Baltimore).

[5] FA MS, KU A, IN L, SH R (2014). Wandering spleen- a diagnostic challenge: case report and review of literature. Malays J Med Sci.

[6] Brown CV, Virgilio GR, Vazquez WD (2003). Wandering spleen and its complications in children: a case series and review of the literature. J Pediatr Surg.

[7] Koliakos E, Papazarkadas X, Sleiman MJ, Rotas I, Christodoulou M (2020). Wandering spleen volvulus: a case report and literature review of this diagnostic challenge. Am J Case Rep.

[8] Draghmeh M, Taher A, Atatri Y, Al-rub FA, Muhaisen W, Khanfar O (2022). Wandering spleen torsion in a patient with polysplenia syndrome. Radiol Case Rep.

[9] Bekheit M, Katri KM, Ezzat T (2012). Wandering hemi-spleen: laparoscopic management of wandering spleen in a case of polysplenia. Int J Surg Case Rep.

[10] Lahiri S, Dasgupta N, Mondal AU (2010). A case of splenic torsion and rupture presenting as ruptured ectopic pregnancy. J Surg Case Rep.

[11] Buehner M, Baker MS (1992). The wandering spleen. Surg Gynecol Obstet.

[12] Graves NS (2013). Acute gastroenteritis. Prim Care.

[13] Sharma A, Salerno G (2014). A torted wandering spleen: a case report. J Med Case Rep.

[14] Singla V, Galwa RP, Khandelwal N, Poornachandra KS, Dutta U, Kochhar R (2008). Wandering spleen presenting as bleeding gastric varices. Am J Emerg Med.

[15] Irak K, Esen I, Keskın M (2011). A case of torsion of the wandering spleen presenting as hypersplenism and gastric fundal varices. Turk J Gastroenterol.

[16] Patil VL, Sasnur P, Sheelin SG (2020). Gastric volvulus with torsion of wandering spleen: a rare case of acute abdomen. J Clin Diagnostic Res.

[17] Tahir F, Ahmed J, Malik F (2020). Post-splenectomy sepsis: a review of the literature. Cureus.

